# Switchable Deep Eutectic Solvents for Lignin Dissolution and Regeneration

**DOI:** 10.3390/polym15214233

**Published:** 2023-10-26

**Authors:** Debao Li, Letian Qi, Mengru Yang, Yujie Gu, Yu Xue, Jiachuan Chen, Ming He, Guihua Yang

**Affiliations:** State Key Laboratory of Biobased Material and Green Papermaking, Qilu University of Technology, Shandong Academy of Sciences, Jinan 250353, China; 10431221053@stu.qlu.edu.cn (D.L.); 10431230284@stu.qlu.edu.cn (M.Y.); 10431230283@stu.qlu.edu.cn (Y.G.); xxyy0707@163.com (Y.X.); heming8916@qlu.edu.cn (M.H.); ygh@qlu.edu.cn (G.Y.)

**Keywords:** lignin, switchable solvent, deep eutectic solvent, ionic liquid

## Abstract

Deep eutectic solvents (DESs) are promising for lignin dissolution and extraction. However, they usually possess high polarity and are difficult to recycle. To overcome this drawback, a variety of switchable ionic liquids (SILs) composed of 1,8-diazabicyclo[5.4.0]undec-7-ene (DBU) and alcohols was synthesized and screened. According to the thermodynamic modeling suggestions, the selected DBU–HexOH SIL was coupled with hydrogen-bond donors to form switchable-DES (SDES) systems with moderated viscosity, conductivity, and pH while maintaining switchability. The SDESs produced a well-improved lignin and lignin model compound solubility compared with those of SILs; charging CO_2_ into SDES (SDES_CO2_) caused a further increase in solubility. The solubility (25 °C) of syringic acid, ferulic acid, and milled wood lignin in SDES_CO2_ reached 230.57, 452.17, and 279.12 mg/g, respectively. Such SDES-dissolved lignin can be regenerated using acetone as an anti-solvent. The SDES-regenerated lignin exhibited a well-preserved structure with no noticeable chemical modifications. Furthermore, the SDES_CO2_ lignin possessed a higher molecular weight (Mw = 10,340 g/mol; Mn = 7672 g/mol), improved uniformity (polydispersity index = 1.35), and a higher guaiacyl lignin unit content compared with the original milled wood lignin. The SDES system proposed in the present work could benefit the fractionation of lignin compounds and facilitate downstream industrial processes.

## 1. Introduction

Abundant reserves of lignocellulosic biomass make it an important renewable resource [1]. As a main component within lignocellulose, lignin is a three-dimensional amorphous polymer composed of methoxylated phenylpropane units [2]. Its aromatic structure causes lignin to be recognized as an important natural source of phenolic chemicals [3]; it is estimated that around 20 billion tons/year of such chemicals are produced by plants [4]. However, the chemical and physical interactions between lignin and carbohydrate components within lignocellulosic biomass make the separation of lignin difficult to achieve [5,6]. Traditional lignin separation approaches, such as chemical treatment, organic solvent treatment, mechanical treatment, and ionic liquid (IL) treatment methods [7], are well developed but, nevertheless, reported to have several drawbacks. Chemical treatment methods, for example, acid or alkali treatment, can achieve lignin separation through the degradation and dissolution of lignin macromolecules [8]; however, their applications are limited by severe lignin deconstruction and unavoidable chemical modifications. In addition, other shortcomings, such as the corrosion of equipment, wastewater treatment, and chemical recycling, also prohibit further application [9]. Organic solvents, such as N-methylmorpholine-N-oxide [10], can be used to dissolve and separate lignin from lignocellulose. However, most of these solvents are toxic and volatile. Because of this, such methods are commonly used for laboratory research and small-scale production [11,12]. Lignin can also be separated through a ball mill treatment [13]; however, this approach is extremely energy-consuming. ILs can selectively remove lignin from the lignocellulose [14], effectively overcoming the lignocellulose obstinacy [12]. Although it is reported to produce lignin with an increased chemical activity [15], the IL method is limited by its high cost [16], poor reusability [17], and, in some cases, it even exhibits greater toxicity than organic solvents [1]. Therefore, it is in the interests of both academic and industrial fields to develop efficient and sustainable methods for lignin dissolution and separation.

A deep eutectic solvent (DES) is a eutectic solvent formulated by hydrogen-bond donors (HBDs) and hydrogen-bond acceptors (HBAs) [18]. ILs can sometimes act as typical HBAs in DES systems [19,20]. Indeed, the DESs share the advantages of ILs, which are low vapor pressure, easy synthesis, and adjustable physical and chemical properties [21]. With the addition of HBDs, a DES can be designed to be low cost [22], with reduced viscosity [23], good biodegradability [24], and low toxicity [25]. In recent years, DESs have been applied in many areas, for example, in organic reactions [26], biotransformations [27], sensor development [28], and pharmaceutical formulation [29]. Furthermore, DESs have been reported as promising solvents for lignocellulosic biomass fractionation due to their selective lignin extraction nature [30,31]. The extracted lignin is usually very pure and uniformly distributed. The selective cleavage of the ether bond in lignin macromolecules during DES treatment has been widely reported [32]. For example, choline chloride–lactic acid DES produced a mild acid-based catalytic environment, which triggered the cleavage of ether bonds between phenylpropane units, resulting in lignin depolymerization and dissolution [33]. The strong hydrogen-bond network within the DES system also facilitated the selective extraction of lignin from the lignocellulose [34,35]. It was reported that acidic DESs with strong HBDs caused the proton-catalyzed cleavage of the ether, ester, and glycosidic linkages present in the lignin–carbohydrate complex, leading to the extraction and depolymerization of lignin and xylan [36,37]. The DES treatment also led to the controllable cleavage of C–O–C and C–C bonds within the lignin macromolecules to produce a lignin stream with improved values [38]. Zhu et al. used a choline chloride–ethylene glycol DES to extract very pure lignin and well-preserved β-O-4 linkages [39]. Xu et al. used an ultrasound-assisted choline chloride–formic acid DES to extract lignin with a small molecular size, narrow distribution, and well-preserved β-O-4 bonds [1]. Although these DES treatments have been proven as efficient lignin dissolution and extraction methods, they still present defects of poor reusability, mainly caused by their high polarities [40,41]. It is, therefore, worth developing a “switchable” DES system, which could act as a polar solvent during the lignin dissolution and extraction processes and could also transfer into a non-polar state during recycling.

**Figure 1:**
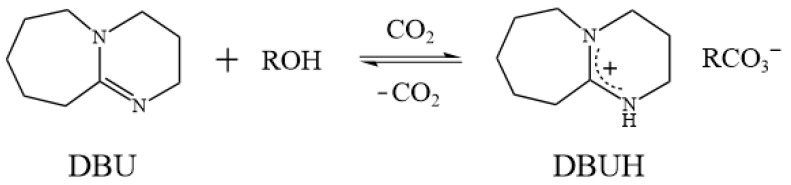
The reaction scheme for the switchable ionic liquids (SILs) [42].

Mixing organic superbases and alcohols, followed by bubbling with CO_2_, generates a so-called switchable ionic liquid (SIL) [42,43], whose chemical and physical properties can be altered by the addition of acid gases, such as CO_2_ and SO_2_ [43]. These SILs can be recycled by reversibly transforming to their molecular precursors by heating or by bubbling N_2_ [44]. Lam Phan et al. found that the exposure of a 1:1 mixture of two neutral liquids, 1,8-diazabicyclo[5.4.0]undec-7-ene (DBU) and monohydric alcohols, to gaseous CO_2_ at 1 atm caused an exothermic conversion of the liquid phase to an ionic-form substance (Figure 1) [42,45]. Anugwom et al. tested the possibility of using these SILs for lignocellulosic biomass treatment [46]. However, like IL defects, SILs are also expensive and highly viscous. Recently, several switchable DESs (SDESs) with a reversible phase transition nature, triggered by CO_2_, pH, and heat, were also developed [47]. Therefore, it would be beneficial to use the concept of DESs, taking SIL as an HBA and coupling it with a selected HBD, to generate a brand-new switchable solvent system, thereby overcoming the defects mentioned previously. A detailed review of the possibility of using switchable solvents for lignin dissolution and extraction was presented in our previous work [48]. The proposed solvent system could provide a low-cost, high-efficiency, and sustainable method for lignin dissolution and extraction; this could further facilitate the development of plant fiber pretreatment technology.

A variety of SILs composed of DBU and different alcohol compounds was synthesized and screened in this work. Optimized SIL was coupled with an HBD to form stable SDES systems; this was followed by using the systems to dissolve lignin and its model compounds. Lignin solubility was checked using ultraviolet spectroscopy, and characterizations such as Fourier-transform infrared (FTIR) spectrum, heteronuclear single-quantum coherence nuclear magnetic resonance (HSQC NMR), gel permeation chromatography (GPC), and thermogravimetric analysis (TGA) were carried out, to confirm the possibility of using the proposed SDES system for lignin dissolution and extraction processes.

## 2. Materials and Methods

### 2.1. Materials

Poplar wood chips were provided by a pulp mill in Shandong, China; 1,8-diazabicyclo[5.4.0]undec-7-ene (DBU, analytical reagent (AR), 99%), ethylene glycol (EG, AR, 98.0%), n-propanol (PrOH, AR, 99.5%), sec-butanol (sBuOH, AR, 99.5%), ethanolamine (ETA, AR, ≥99.0%), decane (AR, 98%), 1,4-dioxane (AR, 99%), tetrahydrofuran (THF, high-pressure liquid chromatography grade, ≥99.9%), syringic acid (AR, 98%), vanillic acid (AR, 98%), syringaldehyde (AR, 98%), ferulic acid (AR, 99%), alkaline lignin (Biopurity), ammonium sulfamate (AR, 99%), imidazole (AR, 99%), dimethyl sulfoxide-d6 (DMSO-d_6_, 99.9%), and potassium bromide (KBr, spectrum pure) were purchased from Shanghai Macklin Biochemical Technology Co., Ltd. (Shanghai, China). Methanol (MeOH, AR, ≥99.5%), glycerol (Gly, AR, ≥99.0%), isopropanol (iPrOH, AR, ≥99.7%), tert-butanol (tBuOH, AR, ≥99.0%), choline chloride (AR, 98.0~101.0%), acetic anhydride (AR, ≥98.5%), and urea (AR, ≥99.0%) were purchased from Sinopharm Chemical Reagent Co., Ltd. (Shanghai, China). Ethanol (EtOH, AR, ≥99.7%), benzene (AR, ≥99.5%), n-hexane (AR, ≥97.0%), and butanol (BuOH, AR, ≥99.5%) were purchased from Tianjin Fuyu Fine Chemical Co., Ltd. (Tianjin, China). Hexanol (HexOH, AR, ≥99.0%), octanol (OctOH, AR, ≥99.0%), and pyridine (AR, ≥99.5%) were purchased from the Tianjin Damao Chemical Reagent Factory (Tianjin, China). Acetone (AR, ≥99.5%) was purchased from the Yantai Far East Fine Chemical Co., Ltd. (Yantai, China). All chemicals were used as received, without further purification.

### 2.2. Preparation of Switchable Ionic Liquids

Switchable ionic liquids (SILs) were synthesized according to the method described in the literature [42,49], where DBU and alcohols (MeOH, EtOH, EG, PrOH, iPrOH, Gly, BuOH, sBuOH, tBuOH, HexOH, OctOH, and ETA) were mixed at a 1:1 molar ratio and stirred at 25 °C. Transparent and uniform SIL systems were obtained, namely, DBU–MeOH, DBU–EtOH, DBU–EG, DBU–PrOH, DBU–iPrOH, DBU–Gly, DBU–BuOH, DBU–sBuOH, DBU–tBuOH, DBU–HexOH, DBU–OctOH, and DBU–ETA. These SILs presented switchable physical and chemical properties upon the absorption or release of CO_2_. According to the method used by [50], polar SIL (SIL_CO2_) can be obtained by bubbling CO_2_ into SIL under room temperature conditions for 30 min until the viscosity of the system increases significantly, whereas non-polar SIL (SIL′) can be restored by heating the SIL_CO2_ at 80 °C for 10 h.

### 2.3. Preparation of Switchable Deep Eutectic Solvents

The switchable deep eutectic solvents (SDES) were synthesized according to the method employed by [51], by using SIL as the hydrogen-bond acceptor (HBA) and water (H_2_O) as the hydrogen-bond donor (HBD). The HBA and HBD were mixed at a 1:10 to 10:1 weight ratio and stirred at 25 °C. A transparent and uniform SDES system was obtained, namely, DBU–HexOH/H_2_O. DBU–HexOH/H_2_O demonstrated switchable physical and chemical properties upon the absorption or release of CO_2_. Polar SDES (SDES_CO2_) can be obtained by bubbling CO_2_ into SDES at room temperature for 30 min until the viscosity of the system increases significantly, whereas non-polar SDES (SDES′) can be restored by heating the SDES_CO2_ at 80 °C for 10 h.

### 2.4. Characterizations of Solvent Properties

The pH of the testing sample was detected using a pH meter (PHS-3G, Shanghai INESA Scientific Instrument Co., Ltd., Shanghai, China) at 25 °C. The conductivity of the testing sample was detected using a conductivity meter (DDS-307A, Shanghai INESA Scientific Instrument Co., Ltd., Shanghai, China). The viscosity of the DES was measured according to the method described in [49], by placing 1 g of sample solution on the fixture of a rotational rheometer (ARES-G2, TA Instruments Vorster, New Castle, DE, USA), operating at 30 rpm. The polarity of the sample to be measured is illustrated by their miscibility in a low polarity reagent (decane) [42].

### 2.5. Preparation of Milled Wood Lignin

The milled wood lignin (MWL) was prepared according to the method recorded in [52]. A specific amount of 80~40 mesh poplar powder was extracted by a solution with a volume ratio of benzene to ethanol of 2:1, and the dried raw material was placed in a planetary ball mill (Pulverisette 5, FRITSCH, Markt Einersheim, Germany). The milled sample was extracted with 1,4-dioxane and deionized water at a volume ratio of 96:4 (*v*/*v*). The extract was centrifuged, and the supernatant was evaporated, concentrated, and freeze-dried to obtain the MWL.

### 2.6. Solubility of the Lignin Model Compound

Lignin solubility was detected according to the method described in [53]. Lignin and its model compounds were added to the testing solvent under continuous stirring at room temperature—25 °C—to fully dissolve until saturation. The liquid phase was separated by filtration using a 0.45 μm organic filter, and the absorption of the dissolved lignin was determined using an ultraviolet-visible spectrophotometer (Agilent Cary 8454, Agilent Technologies, Santa Clara, CA, USA). The lignin concentration was then calculated using Lambert–Beer’s law. The characteristic absorption peaks for lignin and lignin model compounds were listed as follows: alkali lignin 280 nm, syringaldehyde 307 nm, vanillic acid 256 nm, syringic acid 265 nm, and ferulic acid 314 nm [54].

### 2.7. Dissolution and Regeneration of Lignin

Lignin dissolution and regeneration were carried out according to the method employed in [9,55]. MWL was added into the testing solvents (1:100, *w*/*w*) under continuous stirring at room temperature. The fully dissolved lignin solution was filtrated with a 0.45 μm organic filter. Acetone was added to the lignin solution (1:10, *v*/*v*) as an anti-solvent and then kept at 4 °C for 12 h to enable lignin regeneration. The precipitate was recovered using centrifugation (8000 rpm, 10 min) followed by filtration and then oven-dried at 105 °C until a constant weight was achieved.

### 2.8. Characterizations of Lignin

The number average molecular weight (Mn), weight average molecular weight (Mw), and polydispersity index (PDI) were measured by gel permeation chromatography (GPC, e2695, Agilent Technologies Inc., Palo Alto, CA, USA) equipped with an Agilent 1200 series high-performance liquid chromatograph (HPLC) and an ultraviolet detector (UV) [34,56]. Acetylated lignin samples (2 mg) were dissolved in THF (1 mL) and filtered through a 0.45 μm filter. The injection volume was 100 μL, and the wavelength of the UV detector was 280 nm. THF was used as the mobile phase under a flow rate of 100 mL/min. A calibration curve was prepared using polystyrene in the range of 1480~1,233,000 g/mol. The thermal oxidative degradation and stability of the lignin samples were subjected to thermogravimetric analysis (TGA SDT650, TA Instruments, Milford, MA, USA) [57]. The lignin samples were placed in aluminum crucibles and tested between room temperature and 800 °C at a heating rate of 10 °C/min under nitrogen conditions. 

The structural characterization of the lignin samples was carried out using Fourier-transform infrared spectroscopy (FTIR, Bruker ALPHA, Ettlingen, Germany) [3]. A total of 1 mg lignin sample was mixed with 100 mg dried KBr, ground, and pressed into tablets. Samples were scanned 32 times over a range of 400~4000 cm^–1^ at a resolution of 4 cm^−1^. Detailed structural characterization was performed using nuclear magnetic resonance (NMR) spectroscopy (HSQC NMR, BRUKER AVANCE III HD 500 M, Karlsruhe, Germany); around 50 mg of lignin was dissolved in 0.5 mL of DMSO-*d*_6_ [58]. NMR spectra of lignin samples were obtained using a Bruker Avance III HD500 MHz spectrometer at a room temperature of 25 °C. The ^1^H-^13^C heteronuclear single-quantum coherence (HSQC) spectral standard pulse sequence was as follows: spectral width ^1^H of 11 ppm with 2048 sampling points; ^13^C spectral width of 190 ppm with 256 data points; 64 scans; and 1 s scan delay. Volume integration of the signals in the HSQC NMR spectra was performed in Bruker Top Spin 2.1 software.

### 2.9. Molecular Simulation

The molecular simulation was conducted using the conductor-like screening model for real solvents (COSMO-RS) model (BIOVIA COSMOtherm 2020, Version 20.0.0, Dassault Systèmes, Paris, France), in which quantum chemical calculations were combined with statistical mechanics to explore the thermodynamic behaviors of the chemicals used in this work.

The structure of DBUH and HexCO_3_ was drawn by Turbomole (BIOVIA TmoleX 2021, Version 21.0.0, Dassault Systèmes, Paris, France). The geometry optimizations were performed at the density functional theory (DFT) level and utilized the BP function with resolution of identity (RI) approximation; a triple-ξ valence polarized basis set (TZVP) was utilized [59,60]. All the other chemicals were obtained from the built-in database. For the COSMO-RS calculations, it was assumed that ILs were set as a mixture of an equimolar composition of cations and anions, and the DESs were set as a mixture of an equimolar composition of HBAs and HBDs [61].

## 3. Results and Discussion

### 3.1. Characterizations of Switchable Ionic Liquids

**Figure 2:**
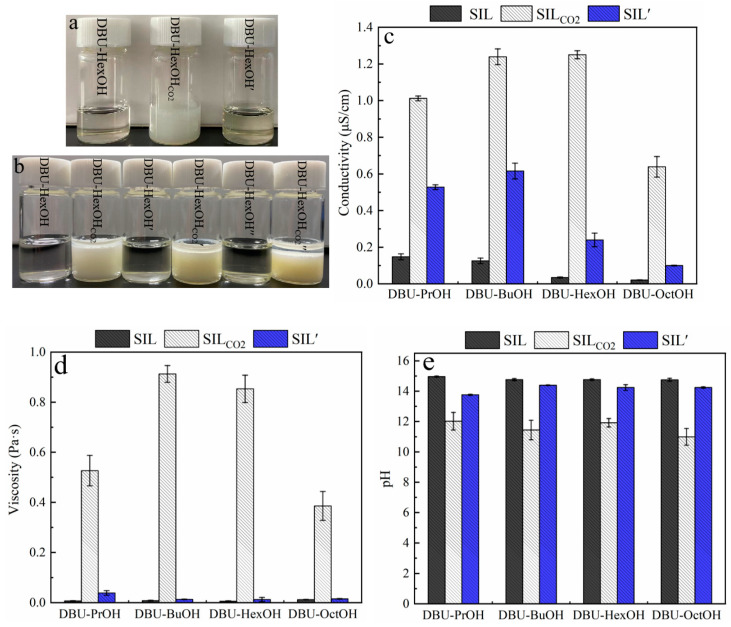
Switchable solvent properties of SILs triggered by CO_2_: (**a**) effect of CO_2_ on the state of DBU–HexOH SIL system; (**b**) effect of CO_2_ on the miscibility of DBU–HexOH in decane; (**c**) effect of CO_2_ on the electric conductivity of DBU-based SILs; (**d**) effect of CO_2_ on viscosity of DBU-based SILs; and (**e**) effect of CO_2_ on pH of DBU-based SILs.

As shown in Figure 2a, a transparent and homogeneous SIL was obtained by simply mixing DBU and monohydric alcohol at room temperature. According to Figure 1, bubbling CO_2_ into SIL caused the formation of its ionic state (SIL_CO2_), leading to a significant increase in viscosity, and sometimes even the formation of solids (Table A1). Among the testing SIL systems, most of the SIL_CO2_ remained as a transparent and homogeneous liquid. For example, DBU–PrOH_CO2_, DBU–BuOH_CO2_, DBU–HexOH_CO2_, and DBU–OctOH_CO2_ were still viscous liquids in the presence of CO_2_. However, DBU–MeOH_CO2_ and DBU–ETA_CO2_ formed rigid solids. The CO_2_ in SIL_CO2_ was removed by heating at 80 °C, after which a transparent SIL’ was obtained. As the presented work focuses on the dissolution and extraction of lignin, the proposed solvent system should maintain the liquid state either with or without the presence of CO_2_. The solvent properties of the SIL system were then investigated; there were notable changes in SIL polarity, conductivity, pH, and viscosity triggered by introducing and removing CO_2_ (Figure 2). 

The polarity of the SIL system can be regulated reversibly by introducing and removing CO_2_. This polarity conversion was verified by mixing SILs with a low-polarity reagent (decane). Taking the DBU–HexOH SIL system as an example, the DBU–HexOH is miscible with decane, whereas its ionic form (DBU–HexOH_CO2_) is not. As shown in Figure 2b, the conversion in polarity remained after three CO_2_ charging–discharging cycles. Pumping CO_2_ through the homogenous SIL–decane solution caused the formation of emulsion, while simply removing CO_2_ by heating restored miscibility. DBU–PrOH, DBU–BuOH, and DBU–OctOH SIL systems (Table A1) demonstrated a similar polarity conversion performance to that of DUB–HexOH. Other SIL systems, however, encountered difficulties in restoring their non-polar state from SIL_CO2_ during the heating process, possibly due to the strong interaction formed between the alcohol compound and CO_2_ [62]. Our study, therefore, focused on the analysis of DBU–PrOH, DBU–BuOH, DBU–HexOH, and DBU–OctOH SIL systems.

The formation of ionic compounds was confirmed by analyzing the conductivity and viscosity of the SIL systems. Generally, SIL and SIL′ demonstrated significantly lower conductivity than that of SIL_CO2_. Alcohol and DBU (0.012 μS/cm) both possess low electroconductivities. As expected, the SILs generated by mixing DBU and alcohols produced low conductivities. The conductivity of SIL, shown in Figure 2c, decreased with the growth of carbon chains, and the conductivity of DBU–PrOH, DBU–BuOH, DBU–HexOH, and DBU–OctOH gradually decreased from 0.148 μS/cm to 0.021 μS/cm. Introducing CO_2_ allowed the SIL to convert into its ionic form, producing a dramatic increase in conductivity in the SIL_CO2_. Among all the tested SIL systems, DBU–HexOH_CO2_ presented the highest conductivity of 1.250 μS/cm. Discharging CO_2_ allowed the electroconductivity to be restored. However, the conductivity of SIL′ was generally higher than that of the SIL system; this could be attributed to the incomplete removal of CO_2_ during the heating process. In terms of their viscosities, SILs presented slightly higher viscosities than those of DBU (0.0005 Pa·s) and alcohols (~10^−4^ Pa·s). As shown in Figure 2d, the viscosities of SILs were within the range of 0.007 Pa·s–0.0126 Pa·s. Similar to conductivity, charging with CO_2_ caused a surge in SIL viscosity, especially for the DBU–BuOH_CO2_ (0.9127 Pa·s) and DBU–HexOH_CO2_ (0.8531 Pa·s) samples. Similarly, viscosity was restored by removing CO_2_ from the system. The significant increase in viscosity relates to the formation of an ionic compound: This created more and stronger ionic interactions, enhancing the internal friction in SIL_CO2_. As shown in Figure 2, the DBU–BuOH and DBU–HexOH SIL systems demonstrated the most notable switching ability for conductivity and viscosity, whereas DBU–BuOH_CO2_ (Figure 2c) presented some difficulties in terms of CO_2_ release when heated. Although there was only a minor difference in their SIL and SIL’ viscosities, the conductivity of DBU–BuOH’ (0.616 μS/cm) was much higher than that of DBU–BuOH (0.126 μS/cm), indicating the incomplete removal of CO_2_. Therefore, DBU–HexOH was deemed the preferred SIL system, as its solvent properties can easily be restored from 1.250 μS/cm to 0.240 μS/cm, demonstrating a preferred switching ability.

DBU is a strong organosuperbase with a pH of 15.01; SILs composed of DBU and alcohols, therefore, also present strong basicity. As shown in Figure 2e, no noticeable variations in the pH of DBU–PrOH, DBU–BuOH, DBU–HexOH, or DBU–OctOH, which ranges from 14.96 to 14.75, could be obtained. Charging with CO_2_ caused the formation of SIL_CO2_ and decreased its pH to around 12, while removing CO_2_ restored the SIL pH to about 14, which is close to that of SIL. Therefore, the SIL system pH was also regulated by charging and discharging CO_2_. For example, DBU–HexOH had a pH of 14.76; charging CO_2_ decreased the pH to 11.92 (DBU–HexOH_CO2_), and removing CO_2_ by heating restored the pH to 14.24 in DBU–HexOH’. However, it is proposed that a strong basicity environment may affect the aromatic structure of lignin, destroy the C–O single bond in lignin, and lead to a significant chemical modification of lignin macromolecules [63]. It would be wise to introduce a cosolvent to moderate the alkaline operating condition, as this may ease the side reactions. In addition, the cosolvent would help reduce the high viscosity of SIL_CO2_, which may prohibit lignin dissolution.

### 3.2. From Switchable Ionic Liquids to Switchable Deep Eutectic Solvents

**Figure 3:**
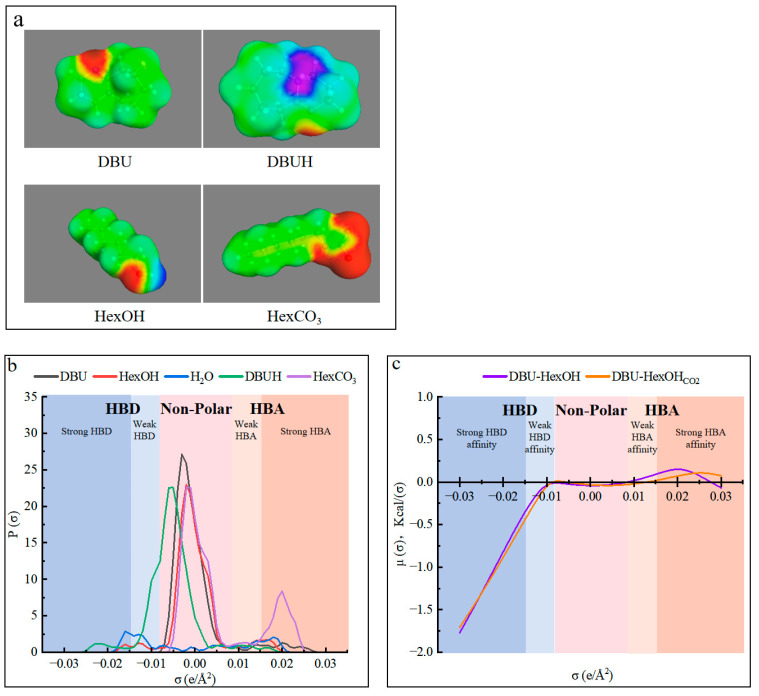
Molecular modeling results from COSMO-RS: (**a**) surface charge distribution for DBU and HexOH before and after charging CO_2_; (**b**) sigma profiles of water and the chemicals in the SIL system; and (**c**) sigma potentials for the DBU–HexOH and DBU–HexOH_CO2_ mixtures.

The use of cosolvent has been reported to enhance the solubility of lignin derivatives [54,64]. However, for this specific use, it should be carefully selected to stabilize the SIL system and promote the hydrogen-bond network within. The molecular simulation was, therefore, conducted with the conductor-like screening model for real solvents (COSMO-RS), in which quantum chemical calculations were combined with statistical mechanics to explore the thermodynamic behavior of the DBU and HexOH used in this work [61]. As was previously demonstrated, charging CO_2_ into SIL caused the formation of ionic SIL_CO2_. The molecule structures and their charge distributions of the chemicals in the DBU–HexOH SIL system are shown in Figure 3. As can be seen in Figure 3a, charging CO_2_ caused the formation of a strong hydrogen-bond donor (HBD) center (blue-purple area) on the DBUH from DBU. In addition, a strong hydrogen-bond acceptor (HBA) zone (red area) was created on the HexCO_3_ from the HexOH. Therefore, in addition to the strong ionic interaction in SIL_CO2_, possible hydrogen bonds could also exist in the DBU–HexOH_CO2_ mixtures. Figure 3b shows the charge distribution on the molecular surface, where the σ values lower than −0.0082 e/Å^2^ are the HBD region, σ values between −0.0082 e/Å^2^ and 0.0082 e/Å^2^ represent the non-polar region, and values higher than 0.00821 e/Å^2^ are the HBA region. As shown in Figure 3b, DBU and HexOH acted as mild polar molecules, as most of their peaks were located in the non-polar and weak HBD (−0.0082~−0.015 e/Å^2^)/weak HBA (0.0082~0.015 e/Å^2^) regions. According to the reaction scheme (Figure 1), charging with CO_2_ converted them into DBUH and HexCO_3_, where a strong HBD peak at −0.021 e/Å^2^ and an HBA peak at 0.020 e/Å^2^ are noticed. The sigma potential results in Figure 3c indicate that only a small change in the mixture was induced by CO_2_ charging, both DBU–HexOH and its ionic form (DBU–HexOH_CO2_) presenting a strong HBD affinity. Due to charging with CO_2_, the formation of ionic substances in SIL_CO2_, therefore, mainly contributes to the internal ionic interactions and only produces a minor effect on the overall hydrogen-bonding ability of the mixture. In addition, based on the COSMO-RS modeling results, introducing a cosolvent such as an HBD is an effective method of enriching the hydrogen-bonding network inside the proposed SIL solvent system. Water normally acts as both an HBD and HBA, presenting characteristic peaks located at −0.016 and 0.017 e/Å^2^, respectively. The addition of water allowed it to perform as an ideal HBD in both the non-ionic (SIL) and ionic (SIL_CO2_) states of the DBU–HexOH system. As shown in Figure 3b, water presented peaks both in the strong (−0.016 e/Å^2^) and weak (−0.013 e/Å^2^) HBD regions, both of which overwhelmed the HBD assignments within the DBU–HexOH and DBU–HexOH_CO2_ (DBUH and HexCO_3_) systems. Therefore, the addition of an HBD as a cosolvent enriched the hydrogen-bond network within the DBU–HexOH SIL system. In this case, the SIL/SIL_CO2_ worked as the HBA and water acted as the HBD within the mixture system.

**Figure A1:**
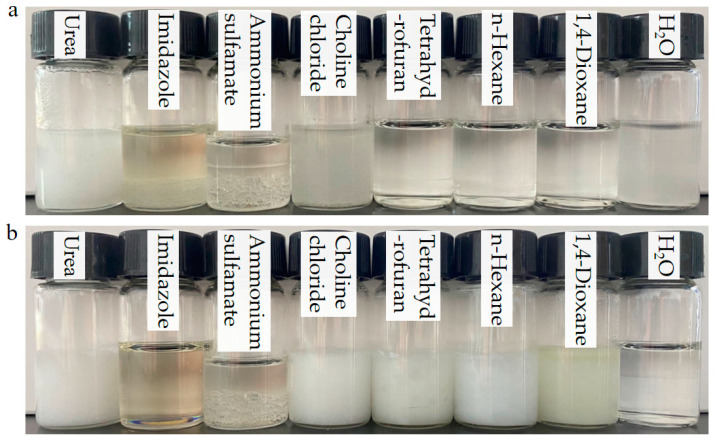
Effect of CO_2_ on the switchable deep eutectics composed of DBU–HexOH and various hydrogen-bond donors: (**a**) DBU–HexOH mixed with various hydrogen-bond donors and (**b**) DBU–HexOH_CO2_ mixed with various hydrogen-bond donors.

**Figure A2:**
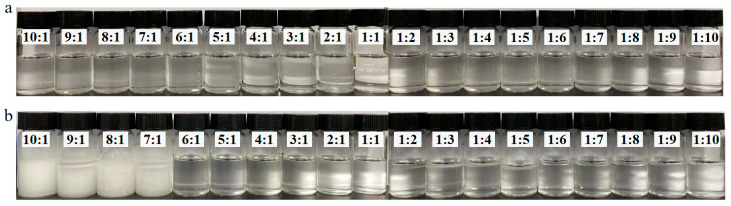
Switchable deep eutectics composed of DBU–HexOH and H_2_O at various proportions (DBU–HexOH/H_2_O (*w*/*w*)): (**a**) DBU–HexOH mixed with H_2_O at various proportions and (**b**) DBU–HexOH_CO2_ mixed with H_2_O at various proportions.

Based on the COSMO-RS thermodynamic modeling results, a switchable deep eutectic solvent (SDES) system was proposed by adding HBDs as cosolvents into the SILs to improve their hydrogen-bonding networks. The addition of the HBD should be able to reduce the viscosity and cost of the solvent, expand the liquid range, and enhance the solubility [36,37]. In this work, SIL and various HBDs were mixed (Figure A1); water was selected as an ideal HBD for the formation of a stable homogeneous and transparent DBU–HexOH/H_2_O system with a 1:5 mass ratio, either with or without the presence of CO_2_ (Figure A2). It can be seen from Table A1 that charging CO_2_ into SDES caused the elevation of conductivity from 4.848 μS/cm (DBU–HexOH/H_2_O) to 23.030 μS/cm (DBU–HexOH/H_2_O_CO2_), and removing CO_2_ allowed the electroconductivity to be restored. However, the conductivity of DBU–HexOH/H_2_O′ (10.066 μS/cm) was higher than that of DBU–HexOH/H_2_O; this could be attributed to the incomplete removal of CO_2_ due to the presence of water. Charging with CO_2_ also increased the viscosity of the SDES system, from 0.0186 Pa·s (DBU–HexOH/H_2_O) to 0.0825 Pa·s (DBU–HexOH/H_2_O_CO2_). Moreover, releasing CO_2_ by heating allowed the viscosity of the DBU–HexOH/H_2_O to be restored to 0.0466 Pa·s. It should be noted that the addition of the HBD significantly reduced the viscosity in the ionic state to about 1/10 of that for the SIL system (0.8531 Pa·s for SIL_CO2_ vs. 0.0825 for SDES_CO2_). In addition, the pH of the DBU–HexOH/H_2_O was 14.26: Charging with CO_2_ decreased the pH to 9.50 (DBU–HexOH/H_2_O_CO2_), which was much lower than that of SIL_CO2_ (DBU–HexOH_CO2_, pH 11.92). Therefore, the proposed SDES, composed of a mixture of DBU–HexOH and water, could moderate the viscosity and pH of the SIL system, while allowing it to maintain its switchable nature, making the SDES more suitable for the lignin dissolution process. 

### 3.3. Lignin Dissolution in Switchable Solvents

The solubility of lignin model compounds in the SIL and SDES systems is shown in Table 1. The DBU–HexOH SIL system demonstrated limited lignin solubility, dissolving 12.12 mg/g syringic acid, 2.01 mg/g vanillic acid, 2.24 mg/g syringaldehyde, 22.87 mg/g ferulic acid, and 10.88 mg/g alkaline lignin at room temperature. However, its ionic form (DBU–HexOH_CO2_) did not demonstrate any lignin solubility. This phenomenon is very similar to that of DBU, which is a major component in SIL. DBU dissolved 22.79 mg/g syringic acid, 4.54 mg/g vanillic acid, 1.17 mg/g syringaldehyde, 2.26 mg/g ferulic acid, and 1.91 mg/g alkaline lignin at room temperature but did not demonstrate lignin solubility after being charged with CO_2_. As the other component in SIL, monohydric alcohol, however, demonstrated a different lignin solubility. Both HexOH and HexOH_CO2_ presented a very small but similar solubility to vanillic acid, syringaldehyde, and ferulic acid. However, charging with CO_2_ increased their solubility for syringic acid, and alkaline lignin increased from 34.9 mg/g and 9.72 mg/g to 40.24 and 16.30, respectively. Although water presented limited lignin solubility and no notable change after charging with CO_2_, the proposed DBU–HexOH/H_2_O SDES system, mixed with SIL and water, demonstrated a notable increase in lignin solubility. The solubility of syringic acid, vanillic acid, syringaldehyde, and ferulic acid in DBU–HexOH/H_2_O achieved 207.58 mg/g, 21.95 mg/g, 7.98 mg/g, and 58.12 mg/g, respectively. These could be further increased by charging CO_2_ into the system. The solubility of syringic acid, vanillic acid, syringaldehyde, and ferulic acid in the DBU–HexOH/H_2_O_CO2_ system was 230.57 mg/g, 78.43 mg/g, 11.64 mg/g, and 452.17 mg/g, respectively. Therefore, the lignin solubility in the SDES system could also be regulated by the addition of CO_2_, and this would vary depending on the type of lignin model compounds. Particularly, for vanillic acid and ferulic acid, after charging CO_2_ into SDES, their solubility increased by 357% and 778%, respectively. However, the solubility of alkaline lignin in SDES decreased from 5.67 mg/g to 4.44 mg/g after forming its ionic state. Therefore, the proposed SDES mixed with DBU–HexOH and H_2_O demonstrated improved lignin solubility, and this was also regulated by charging and discharging CO_2_.

The proposed SDES system was further tested for the dissolution of milled wood lignin (MWL). The DBU–HexOH SIL dissolved 9.05 mg/g MWL, while the DBU–HexOH/H_2_O SDES dissolved up to 213.35 mg/g MWL. Charging CO_2_ into the SDES further increased MWL solubility to 279.12 mg/g. The SDES-dissolved lignin was easily regenerated using acetone as an anti-solvent, with a yield of 84.31% (SDES_CO2_–MWL). The variations in weight average molecular weight (Mw), number average molecular weight (Mn), and the polydispersity index (PDI) before and after SDES treatment were tested using gel permeation chromatography (GPC); the results are given in Table 2. In comparison with the MWL lignin, both regenerated lignin samples demonstrated a higher molecular weight. The native lignin sample (MWL) was 7701 g/mol Mw and 2959 g/mol Mn, with a PDI of 2.60. The SDES-treated MWL sample had a similar PDI to that of its raw material (MWL), but the regenerated lignin demonstrated an elevated molecular weight of 9823 g/mol Mw and 3397 g/mol Mn. SDES_CO2_ treatment resulted in a lignin stream with a much higher molecular weight (Mw = 10,340 g/mol, Mn = 7672 g/mol) and with an improved uniformity (PDI = 1.35). Compared with the traditional DES–lignin (Table 2), the lignin stream produced in this work had a well-preserved long-chain structure and a much higher molecular weight. Therefore, the GPC results indicate that the SDES treatment is a promising method for lignin dissolution and extraction, as it not only regulated the lignin solubility with CO_2_ but also produced a lignin stream with a high molecular weight and improved uniformity, both of which may benefit the downstream process. 

**Figure 4:**
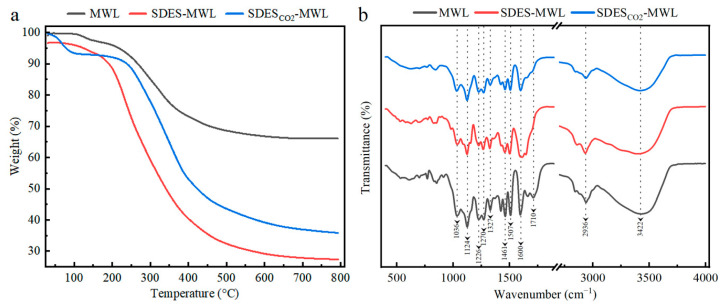
Thermogravimetric analysis (**a**) and Fourier-transform infrared spectroscopy (**b**) of lignin samples.

The thermal oxidative degradation and stability of the MWL before and after SDES treatments were investigated using the thermogravimetric analysis (TGA) [65]. As shown in Figure 4a, all the testing samples exhibited a similar thermal performance, divided into three stages. The first stage was the initial degradation stage (80~200 °C), most likely caused by the removal of moisture and volatile components [66]. The second stage was the main degradation stage of lignin (200~400 °C), where lignin degradation occurred. Carboxylation breakdown of aliphatic hydroxyl groups and ether bonds present in the structure of lignin occurred, while degradation can be attributed to the side chain dehydrogenation reaction [67]. Both SDES–MWL and SDES_CO2_–MWL samples demonstrated a more significant mass loss, which is possibly due to the partial breakage of connections between lignin macromolecules after regeneration. The third stage was the carbonization stage (400~600 °C), where methoxy groups and C–C bonds of lignin were disrupted, with the release of volatiles and production of bio-oils [68]. The decomposition of the lignin sample in Figure 4a occurred slowly at this stage, resulting in char residue [69]. This could be attributed to the production of highly branched and extremely condensed aromatic structures. The MWL sample produced a high char residue rate of 66.7%; this could be attributed to the fact that the sample had a highly complex and condensed lignin structure. MWL is easily converted to char residue owing to its structural resemblance [68]. Once the MWL had reached a temperature of 600 °C, the quality of the coke was stable and no further reaction occurred. However, both SDES- and SDES_CO2_-treated MWL produced lower char residue rates: 28.3% and 1.3%, respectively. This indicates the lower thermal stability of SDES–MWL and SDES_CO2_–MWL compared with that of the MWL sample. 

Structural characterization of lignin before and after SDES treatment was performed using Fourier-transform infrared (FTIR) spectroscopy; the assignments of the characterizing peaks are shown in Table A2. As shown in Figure 4b, the stretching vibration for hydroxyl is observed at 3422 cm^−1^ [70]. The stretching vibrations for the lignin aromatic ring skeleton at 1600 cm^−1^ and 1507 cm^−1^ can be seen in all testing samples; this indicates that no significant change in the aromatic ring structure of MWL occurred during the SDES treatments [34,71]. The peak at 2936 cm^−1^ was attributed to the C–H vibration in CH_3_-/CH_2_ = groups. The characteristic peaks at the wave number of 1461 cm^−1^ were assigned to the C–H asymmetric vibration of CH_2_ = groups and C–H transformation in the aromatic rings [3]. All the characteristic peaks mentioned above were found in all the testing samples. Stretching vibrations for C=O bonds are typically found between 1740 and 1700 cm^–1^; this is where signals attributable to C=O bonds in unconjugated ketones, carbonyls, and ester groups are normally observed. Lower absorption energies (around 1700 cm^–1^) were reported for conjugated aldehydes and carboxyl acids [72]. MWL produced a characteristic peak at 1710 cm^–1^, while SDES–MWL and SDES_CO2_–MWL demonstrated lower intensities within this range, indicating lower carbonyl and carboxyl group content. The peak at 1327 cm^−1^ was attributed to the stretching vibration of the C–O in the syringyl (S) lignin unit, whereas the peaks at 1270 cm^−1^ represent the stretching vibration of the C–O in the guaiacyl (G) lignin unit [73]. The peak at 1124 cm^−1^ demonstrates the presence of syringyl moieties in lignin because it represents the C–H deformation of the lignin S unit. Therefore, a feature of GS-type lignin was demonstrated in the FTIR spectra. Additionally, the signal at 1036 cm^−1^, corresponding to the C–O deformation of primary –Ohs, was detected in all lignin fragments [74]. The FTIR spectra did not show any noticeable chemical modifications occurring in MWLs during the SDES treatments.

**Figure 5:**
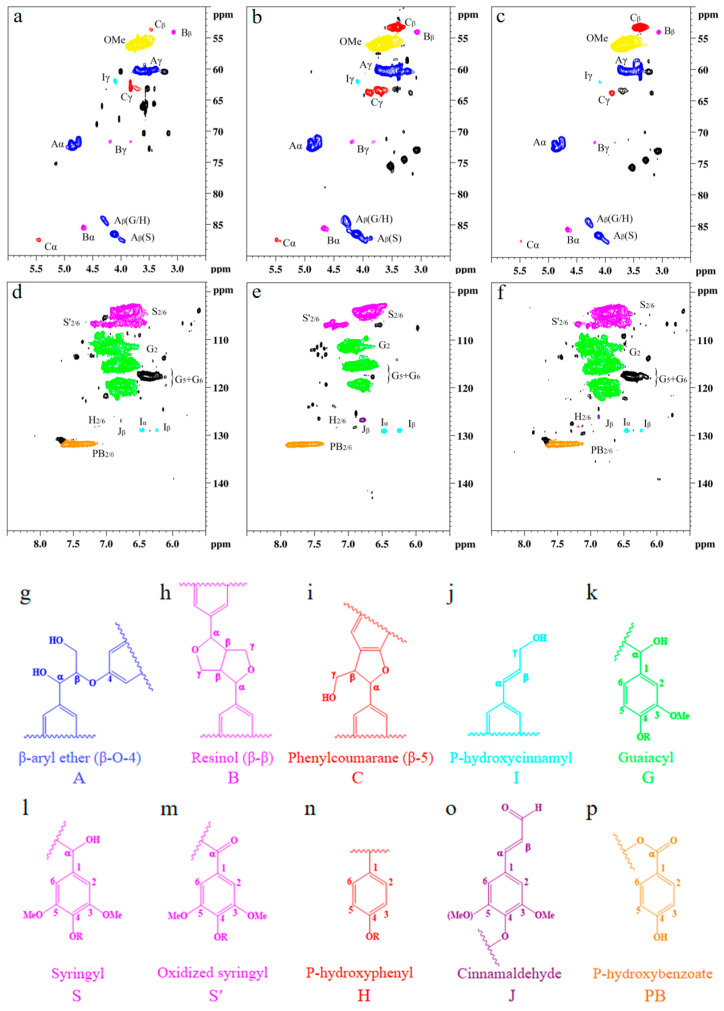
Nuclear magnetic resonance analysis of lignin with or without treatment: (**a**) side-chain regions of the milled wood lignin; (**b**) side-chain regions of SDES-treated lignin; (**c**) side-chain regions of the SDES_CO2_-treated lignin; (**d**) aromatic regions of the milled wood lignin; (**e**) aromatic regions of the SDES-treated lignin; (**f**) aromatic regions of the SDES_CO2_-treated lignin; (**g**) β-O-4 aryl ethers; (**h**) resinol; (**i**) phenylcoumarane; (**j**) p-hydroxycinnamyl alcohol end group; (**k**) guaiacyl unit; (**l**) syringyl unit; (**m**) oxidized (Cα = O) syringyl unit; (**n**) p-hydroxyphenyl unit; (**o**) cinnamaldehyde end group; and (**p**) p-hydroxybenzoate. The abbreviations A, B, C, I, G, S, S’, H, J, and PB listed in (**g**–**p**) are used to indicate the different types of lignin structures shown in (**a**–**f**).

Detailed structural analysis of MWL and SDES–MWL and SDES_CO2_–MWL was characterized using 2D HSQC NMR spectroscopy [75,76]; the side chain (δ_C_/δ_H_ 50–90/2.5–6.0) and the aromatic (δ_C_/δ_H_ 100–135/5.5–8.5) regions of the lignin samples are shown in Figure 5, and the main lignin cross-signal assignments are listed in Table A3. The related signals for β-aryl ether ethers (A), resin alcohols (B), and phenylcoumaranes (C) in the lignin samples were found in all the testing samples, indicating a well-preserved lignin structure after treatment. The peaks corresponding to β-aryl ether connections are shown for A_α_ (δ_C_/δ_H_ 71.8/4.86 ppm), A_γ_ (δ_C_/δ_H_ 59.5–59.7/3.40–3.63 ppm), A′_γ_ (δ_C_/δ_H_ 63.2/4.33–4.49 ppm), A_β(G/H)_ (δ_C_/δ_H_ 83.9/4.29 ppm), and A_β(S)_ (δ_C_/δ_H_ 85.9/4.12 ppm). β-β connections are shown for B_α_ (δ_C_/δ_H_ 84.8/4.65 ppm), B_β_ (δ_C_/δ_H_ 53.5/3.06 ppm), and B_γ_ (δ_C_/δ_H_ 71.0/3.82 and 4.18 ppm). β-5 connections are shown for C_α_ (δ_C_/δ_H_ 86.8/5.46 ppm), C_β_ (δ_C_/δ_H_ 53.3/3.46 ppm), and C_γ_ (δ_C_/δ_H_ 62.5/3.73 ppm). Moreover, methoxyl connections were found at δ_C_/δ_H_ 55.6/3.73 ppm. The signals for the C_α_–H_α_, C_β_–H_β_, and C_γ_–H_γ_ correlations of substructures (I) were seen clearly at δ_C_/δ_H_ 128.4/6.44, 128.2/6.25, and 61.4/4.10, respectively. It was found that all the lignin samples exhibited similar spectral patterns. Three main types of linkages, namely, aromatic ether bond β-O-4, β-β in resin, and β-5 in phenylcoumarane [73], were observed in all testing lignin samples, with only minor changes in their distributions. The MWL produced β-O-4 linkages of 40.71/100Ar, β-β linkages of 2.71/100Ar, and β-5 linkages of 3.57/100Ar cross-peaks; the SDES–MWL produced β-O-4 linkages of 38.29/100Ar, β-β linkages of 3.76/100Ar, and β-5 linkages of 4.03/100Ar; finally, the SDES_CO2_–MWL produced β-O-4 linkages of 38.27/100Ar, β-β linkages of 3.37/100Ar, and β-5 linkages of 3.63/100Ar. The main linkages within lignin macromolecules, therefore, remained unchanged after the SDES and SDES_CO2_ treatments, which agrees with the FTIR results. In addition, all the lignin samples exhibited strong signals for methoxy moieties, indicating that the –OCH_3_ groups also remained unchanged after the SDES and SDES_CO2_ treatments. 

In the aromatic region, the C_2,6_–H_2,6_ correlations from S-type units and the sum of C_2_–H_2_, C_5_–H_5_, and C_6_–H_6_ correlations from G-type units were used to estimate the S/G ratio [74]. A strong signal at δ_C_/δ_H_ 103.8/6.71 belonged to the S unit at C_2,6_–H_2,6_, whereas the G units showing the cross-peak signals for C_2_–H_2_ (δ_C_/δ_H_ 110.9/6.98), C_5_–H_5_ (δ_C_/δ_H_ 114.9/6.77), and C_6_–H_6_ (δ_C_/δ_H_ 119.0/6.80) can be found in all the lignin samples. It can, therefore, be confirmed that the MWL used in this work was predominantly comprised of S and G units. The S/G ratios of MWL, SDES–MWL, and SDES_CO2_–MWL were 0.88, 0.73, and 0.76, respectively, indicating that the SDES and SDES_CO2_ treatments caused slightly more S-type lignin loss than G-type lignin loss. Other signals observed in the aromatic regions could be assigned to p-hydroxycinnamyl alcohol end groups (I), cinnamaldehyde end groups (J), and p-hydroxybenzoate substructures (PB). The C–H correlated signals for I and J were found at δ_C_/δ_H_ 128.2/6.44 and 126.1/6.76 ppm, respectively. The C_2,6_–H_2,6_ correlations for PB were observed as a strong signal at δ_C_/δ_H_ 131.2/7.67. These characterization results clearly demonstrate, again, that the lignin macromolecule framework remained intact following both the SDES and SDES_CO2_ treatments. 

## 4. Conclusions

In this work, switchable ionic liquids (SILs) composed of DBU and different alcohols were screened, based on their solvent properties. DBU–HexOH was selected as the optimized SIL for its stable liquid state and notable switchable nature in conductivity, viscosity, and pH, created by the addition and removal of CO_2_. According to the thermodynamic molecular modeling results, the selected SIL was coupled with a hydrogen-bond donor (HBD) to form a switchable deep eutectic solvent (SDES) system, providing moderate viscosity and pH while maintaining its switchable nature. The DBU–HexOH SIL system resulted in a switchable lignin dissolution performance, which was regulated by charging and discharging CO_2_. The addition of water as an HBD improved lignin solubility in the SDES, while preserving its switchable nature. The DBU–HexOH SIL dissolved 9.05 mg/g MWL, while the DBU–HexOH/H_2_O SDES dissolved up to 213.35 mg/g MWL. Charging CO_2_ into the SDES further increased MWL solubility to 279.12 mg/g. The lignin dissolved in SDES systems can be regenerated using acetone as an anti-solvent. Both FTIR and HSQC results indicate that the SDES- and SDES_CO2_-treated lignin did not produce any notable chemical modifications compared to the MWL sample. Interestingly, the regenerated lignin treated by SDES_CO2_ produced a high molecular weight (Mw = 10,340 g/mol; Mn = 7672 g/mol) and improved uniformity (PDI = 1.35). On the other hand, the original MWL was only 7701 g/mol Mw and 2959 g/mol Mn, with a PDI of 2.60. Therefore, the presented work proposed a notable SDES system, whose solvent properties can be regulated by the charging and discharging of CO_2_. Moreover, it can be used for lignin dissolution and extraction to produce a lignin stream with a well-preserved structure, high molecular weight, and improved uniformity, all of which may facilitate the downstream process.

## Figures and Tables

**Table 1:** Solubility of switchable solvent system to lignin model compounds (25 °C, mg/g).

	Syringic Acid	Vanillic Acid	Syringaldehyde	Ferulic Acid	Alkaline Lignin
H_2_O	8.20 ± 3.17	0.63 ± 0.01	0.52 ± 0.01	0.28 ± 0.28	0.88 ± 0.01
H_2_O_CO2_	6.12 ± 0.20	0.55 ± 0.01	0.99 ± 0.01	0.60 ± 0.01	1.44 ± 0.01
DBU	22.79 ± 0.01	4.54 ± 0.85	1.17 ± 0.01	2.26 ± 0.01	1.91 ± 0.01
DBU_CO2_	-	-	-	-	-
HexOH	34.80 ± 0.12	0.04 ± 0.01	3.12 ± 0.01	0.04 ± 0.01	9.72 ± 0.07
HexOH_CO2_	40.24 ± 0.01	0.04 ± 0.01	4.03 ± 0.01	0.03 ± 0.01	16.30 ± 0.12
DBU–HexOH	12.12 ± 0.08	2.01 ± 0.01	2.24 ± 0.01	22.87 ± 1.46	10.88 ± 0.02
DBU–HexOH_CO2_	-	-	-	-	-
DBU–HexOH/H_2_O	207.58 ± 0.07	21.95 ± 0.03	7.98 ± 0.02	58.12 ± 14.33	5.67 ± 0.04
DBU–HexOH/H_2_O_CO2_	230.57 ± 0.12	78.43 ± 0.18	11.64 ± 0.03	452.17 ± 1.42	4.44 ± 0.04

Note: - Data with extremely low solubility and which could not be detected.

**Table 2:** Molecular weights and polydispersity indices of lignin.

	Mw (g/mol)	Mn (g/mol)	PDI
Lit. MWL *	6374	5542	1.15
CCL–lignin *	4416	2349	1.88
CLL–lignin *	1805	971	1.86
MWL	7701	2959	2.60
SDES–MWL	9823	3397	2.89
SDES_CO2_–MWL	10,340	7672	1.35

Note: * Data from Liu et al. [34].

## Data Availability

The data presented in this study are available upon request from the corresponding author.

## Appendix A

**Table A1:** Solvent properties of the proposed switchable ionic liquids and switchable deep eutectics.

SIL/SDES	pH	Conductivity (μS/cm)	Viscosity (Pa·s)	Polarity
DBU–MeOH	15.14 ± 0.16	0.307 ± 0.089	0.1095 ± 0.0972	Non-polar
DBU–MeOH_CO2_	-	-	-	Polar
DBU–MeOH′	14.48 ± 0.21	0.863 ± 0.161	0.0205 ± 0.1590	Polar
DBU–EtOH	15.08 ± 0.20	0.224 ± 0.037	0.1337 ± 0.1894	Non-polar
DBU–EtOH_CO2_	12.48 ± 0.56	1.075 ± 0.365	0.6755 ± 0.3039	Polar
DBU–EtOH′	14.58 ± 0.12	0.681 ± 0.003	0.0190 ± 0.0022	Polar
DBU–EG	14.32 ± 0.02	0.112 ± 0.010	0.0474 ± 0.0007	Polar
DBU–EG_CO2_	12.83 ± 0.17	0.278 ± 0.024	2.0262 ± 0.0322	Polar
DBU–EG′	13.25 ± 0.37	0.690 ± 0.245	0.2110 ± 0.0007	Polar
DBU–PrOH	14.96 ± 0.04	0.148 ± 0.016	0.0070 ± 0.0013	Non-polar
DBU–PrOH_CO2_	12.02 ± 0.58	1.012 ± 0.013	0.5267 ± 0.0607	Polar
DBU–PrOH′	13.76 ± 0.04	0.528 ± 0.013	0.0389 ± 0.0090	Non-polar
DBU–PrOH	15.01 ± 0.11	0.195 ± 0.011	0.1734 ± 0.1444	Non-polar
DBU–iPrOH_CO2_	12.62 ± 0.93	1.066 ± 0.012	0.4049 ± 0.0453	Polar
DBU–iPrOH′	14.33 ± 0.09	0.402 ± 0.006	0.0296 ± 0.2647	Polar
DBU–Gly	13.95 ± 0.35	0.063 ± 0.041	0.5086 ± 0.0017	Polar
DBU–Gly_CO2_	13.27 ± 1.09	0.158 ± 0.061	1.2097 ± 0.0047	Polar
DBU–Gly′	13.68 ± 0.23	0.163 ± 0.067	1.0338 ± 0.0145	Polar
DBU–BuOH	14.76 ±0.07	0.126 ± 0.015	0.0085 ± 0.0015	Non-polar
DBU–BuOH_CO2_	11.44 ± 0.64	1.239 ± 0.043	0.9127 ± 0.034	Polar
DBU–BuOH′	14.39 ± 0.02	0.616 ± 0.043	0.0138 ± 0.0009	Non-polar
DBU–sBuOH	14.94 ± 0.05	0.047 ± 0.010	0.0057 ± 0.0019	Non-polar
DBU–sBuOH_CO2_	13.01 ± 0.14	0.799 ± 0.039	0.3782 ± 0.0769	Polar
DBU–sBuOH′	14.38 ± 0.10	0.328 ± 0.259	0.0173 ± 0.0008	Non-polar
DBU–tBuOH	14.75 ± 0.30	0.018 ± 0.014	0.0005 ± 0.0003	Non-polar
DBU–tBuOH_CO2_	13.49 ± 0.26	0.252 ± 0.047	0.3252 ± 0.1020	Polar
DBU–tBuOH′	14.36 ± 0.46	0.202 ± 0.098	0.0458 ± 0.0012	Non-polar
DBU–HexOH	14.76 ± 0.05	0.035 ± 0.003	0.0060 ± 0.0019	Non-polar
DBU–HexOH_CO2_	11.92 ± 0.28	1.250 ± 0.022	0.8531 ± 0.055	Polar
DBU–HexOH′	14.24 ± 0.19	0.240 ± 0.037	0.0131 ± 0.0080	Non-polar
DBU–OctOH	14.75 ± 0.10	0.021 ± 0.001	0.0126 ± 0.0007	Non-polar
DBU–OctOH_CO2_	10.99 ± 0.55	0.639 ± 0.056	0.3857 ± 0.0576	Polar
DBU–OctOH′	14.24 ± 0.05	0.100 ± 0.002	0.0156 ± 0.0011	Non-polar
DBU–ETA	14.91 ± 0.05	0.100 ± 0.017	0.0116 ± 0.0007	Polar
DBU–ETA_CO2_	-	-	-	-
DBU–ETA′	-	-	-	-
DBU–HexOH/H_2_O	14.26 ± 0.28	4.848 ± 0.422	0.0186 ± 0.0011	Polar *
DBU–HexOH/H_2_O_CO2_	9.50 ± 0.60	23.030 ± 3.010	0.0825 ± 0.0205	Polar *
DBU–HexOH/H_2_O′	11.19 ± 0.50	10.066 ± 2.854	0.0466 ± 0.0046	Polar *

Note: - Data that cannot be determined as the sample is solid; * Data test with the presence of water.

**Table A2:** Assignment of Fourier-transform infrared spectrum analysis of lignin samples.

Wavenumbers (cm^−1^)	Assignment (Bond)	MWL	SDES-MWL	SDES_CO2_-MWL
3422	O–H stretching vibration	√	√	√
2936	C–H stretching vibration in methyl	√	√	√
1710	C=O stretching vibration	√	×	×
1600, 1507	Aromatic ring skeleton vibration	√	√	√
1461	C–H deformation vibration in –CH_2_–	√	√	√
1373	C–H bending vibration of aliphatic compounds in carbohydrate	√	×	×
1126, 1327	C–O stretching vibration of syringyl units	√	√	√
1270	C–O stretching vibration of guaiacyl units	√	√	√
1124	C–H stretching vibration of syringyl units	√	√	√
1036	C–H bending vibration of guaiacyl units	√	√	√

**Table A3:** Assignments of ^13^C-^1^H correlation signals in the HSQC NMR spectra of MWL and SDES-MWL and SDES_CO2_-MWL.

Labels	δ_C_/δ_H_ (ppm)	Assignment
C_β_	53.3/3.46	C_β_−H_β_ in phenylcoumarane substructures (C)
B_β_	53.5/3.06	C_β_−H_β_ in resinol substructures (B)
–OCH_3_	55.6/3.73	C−H in methoxyls
A_γ_	59.5-59.7/3.40-3.63	C_γ_−H_γ_ in β-O-4′ substructures (A)
I_γ_	61.4/4.10	C_γ_−H_γ_ in p-hydroxycinnamyl alcohol end groups (I)
A′_γ_	63.2/4.33-4.49	C_γ_−H_γ_ in γ-acylated β-O-4′ substructures (A′)
C_γ_	62.5/3.73	C_γ_−H_γ_ in phenylcoumarane substructures (C)
B_γ_	71.0/3.82 and 4.18	C_γ_−H_γ_ in resinol substructures (B)
A_α_	71.8/4.86	C_α_−H_α_ in β-O-4′ substructures (A)
A_β(G/H)_	83.9/4.29	C_β_−H_β_ in β-O-4′ substructures linked to G/H units (A)
B_α_	84.8/4.65	C_α_−H_α_ in resinol substructures (B)
A_β(S)_	85.9/4.12	C_β_−H_β_ in β-O-4′ substructures linked to S units (A)
C_α_	86.8/5.46	C_α_−H_α_ in phenylcoumarane substructures (C)
S_2,6_	103.8/6.71	C_2,6_−H_2,6_ in etherified syringyl units (S)
S′_2,6_	106.2/7.23 and 7.07	C_2,6_−H_2,6_ in oxidized (Cα = O) syringyl units (S′)
G_2_	110.9/6.98	C_2_−H_2_ in guaiacyl units (G)
G_5_	114.9/6.77	C_5_−H_5_ in guaiacyl units (G)
G_6_	119.0/6.80	C_6_−H_6_ in guaiacyl units (G)
I_β_	128.2/6.25	C_β_−H_β_ in p-hydroxycinnamyl alcohol end groups (I)
I_α_	128.4/6.44	C_α_−H_α_ in p-hydroxycinnamyl alcohol end groups (I)
PB_2,6_	131.2/7.67	C_2,6_−H_2,6_ in *p*-hydroxybenzoate substructures (PB)
J_β_	126.1/6.76	C_β_−H_β_ in cinnamaldehyde end groups (J)
H_2,6_	127.9/7.19	C_2,6_−H_2,6_ in p-hydroxyphenyl units (H)
